# Multiplex immunohistochemistry/immunofluorescence is superior to tumor mutational burden and PD‐L1 immunohistochemistry for predicting response to anti‐PD‐1/PD‐L1 immunotherapy

**DOI:** 10.1111/1759-7714.13233

**Published:** 2019-11-12

**Authors:** Weixian Hu

**Affiliations:** ^1^ Department of General Surgery Guangdong Provincial People's Hospital, Guangdong Academy of Medical Sciences Guangzhou China

Since cancer was first reported, the fight against it has been ongoing. In 1971, scientists formally claimed to have found a cure for cancer and multiple breakthroughs have subsequently been made.^1^ However, since then, nearly half a century has elapsed and cancer still remains one of the leading threats to human health. The main problem is that we have tried to use various shortcuts to fight cancer without properly understanding it. However, although our treatments are constantly evolving, so are the cancers.^2^ We began by surgically removing tumors and then continued treatment by using chemotherapy, radiotherapy, and other treatment strategies to improve post‐surgical survival. But as the number of surgically unresectable tumors increased, these externally prescribed therapies, alone or in combination, were given prior to surgery in an attempt to shrink the tumor, converting unresectable tumors to surgically resectable. Then, based on the perioperative or post‐surgical pathology observations, doctors evaluated their potential adjuvant efficacies to determine whether they should be prescribed further adjuvant therapy. The main problem at present is that despite the use of all these treatment modalities, cancers are not only still recurring after treatment but they are also able to resist previous treatments which worked.^3^

Over time, we have devoted a plethora of efforts in treating cancer from external sources, such as prescribing drugs or radiation, until we turned our attention to looking internally by innovative means, whereby we have started to inform our immune system about the harmful cancerous invaders that tricked the immune effector cells into believing they were bodily residents, which is known as immunoediting.^4^ As such, immunotherapy aims to harness and arm our own immune system to better recognize these invaders.^5^ However, since its clinical application, we have not only found that the responses are not as promising in all cancers, but are often also accompanied by severe immune‐related adverse events (irAEs), which at times can be fatal.^6^ Researchers are now investigating using a combination of immunotherapy with other treatment modalities^7^, ^8^ but the main obstacle has been a long‐existing problem that we have always faced: finding biomarkers able to predict treatment response and at their best, stratifying respondents from nonrespondents to better match patients to treatments which would provide the most beneficial quality of life and a lower financial burden without jeopardizing treatment and survival outcomes.

In a recently published study by Yu *et al*. the authors merged the findings of 24 reports on the most common tissue‐based biomarkers, namely programmed cell death ligand 1 (PD‐L1) immunohistochemistry (IHC), 10 on tumor mutational burden (TMB), nine on gene expression profiling (GEP), seven on multiplex immunohistochemistry/ immunofluorescence (mIHC/IF) and multimodality assays.^9^ A total of 8135 patients with more than 10 different solid tumors were analyzed to ascertain which one would better predict treatment response to anti‐PD‐1/PD‐L1 therapy. Previous studies have suggested that when the area under the curve (AUC) of the detection method is ≥0.80, it can be considered as reliable. In this study, the authors found that the prediction accuracy of mIHC/IF (weighted/unweighted AUC, 0.790 or 0.872; 95% confidence interval [CI], 0.650–0.688 or 0.657–0.710) was higher than other biomarkers. Additionally, they found that mIHC/IF was least likely prone to false‐positive results as it had significantly higher positive predictive values and positive likelihood ratios (LR+). In contrast, despite PD‐L1 IHC being widely used clinically to predict the therapeutic response to anti‐PD‐1/PD‐L1 therapy, comparatively it has the lowest AUC and poor LR. The inferior performance of GEP to mIHC/IF could be because of spatial and coexpression assessments, related to the PD‐1 and PD‐L1 proximity, the CD8+ cell density and other potential nonaccountable variables in the investigated GEP assays. TMB was found to be more suitable for predicting the prognosis of the less inflamed tumor microenvironment (TME). The authors demonstrated that PD‐L1 IHC in combination with TMB had higher AUC and LR+ than when they were used separately. Recently, most of the biomarkers that have been discovered have been mainly used to identify patients who do not respond to anti‐PD‐1/PD‐L1treatment. However, the authors found that mIHC/IF could identify patients who would respond, and not respond, to anti‐PD‐1/PD‐L1 therapy.

mIHC/IF combines the advantages of IHC, IF and flow cytometry to preserve signal amplification and spatial relationships by performing multi‐antigen tissue staining, allowing the visualization of cell‐cell interaction in TME. This is a new technique designed to reveal hidden genomic alterations in tissue samples.^10^ It allows automated segmentation to distinguish between tumoral and nontumoral tissues, and can ensure assay reproducibility; making it of great significance for clinical application.^11^ Therefore, the findings of this study are striking as they found that the commonly used method, PD‐L1 IHC, was not as effective as previously thought and that mIHC/IF provided important understandings about the spatial tumor‐immune interactions and protein biomarker coexpression by demonstrating the superior stratifying capability for differentiating between respondents and nonrespondents. This means that mIHC/IF has a higher predictive accuracy, can reduce the risk of nonresponders’ exposure to irAEs, provide timely treatment to these patients and improve the cost of treatment.

Although immunotherapy is increasingly being used in clinical practice, immunotherapy can be viewed as a treatment approach that has not yet attained its full potential.^12^, ^13^ Considering the heterogeneity existing between populations, patients, and cancers themselves, simply aiming to find only one biomarker or factor to distinguish between respondents and nonrespondents nowadays seems a far‐fetched concept.

Based on actual understanding, an optimal approach might be to use a clinicoradiogenomic model to accelerate the progress of identifying novel biomarkers to enhance the stratification of patients into more homogeneous groups for investigating the treatment to which they would be most responsive and from which they would receive the greatest benefits. For this, first we can analyze the patients’ data by combining and permuting their clinicopathological characteristics to find the best homogenous approach in which to group them. Second, we can use radiomics to phenotype the distinguishing imaging and pathological characteristics of their pre‐ and post‐treatment disease to better regroup them. Third, using genomics to decipher the patients’ bodily and cancerous environment, we can further optimize the re‐classification of these patients into a more homogeneous cohort. This may appear to be arduous research but with the help of artificial intelligence the process can be accelerated and standardized, and the benefits obtained could surpass the initial difficulties of translating laboratory findings into clinically applicable therapies.^14^ Based on these, we may be able to enhance personalized cancer treatment, or at the very least, have a better understanding of the tumor, host, and microenvironment. For this, global data sharing would be an important milestone to attain.^15^

mIHC/IF has a more superior predictive accuracy than PD‐L1 IHC, GEP and TMB, and could become a benchmark for differentiating between responders and nonresponders to anti‐PD‐1/PD‐L1 therapy. At present, scientists are keen to develop novel and more effective biomarkers to improve the effectiveness of cancer immunotherapy. However, the focus should not be solely on identifying or validating them, but also on optimizing ways to use them in order to deepen our understanding of the underlying metabolic cascade leading to cancer evolution.

## Disclosure

The author declares no competing interests.
